# Beyond Comorbidity: Evolutionary Insights Into the Concomitance of Neurodivergence, Major Depressive Disorder, and Anxiety Disorders

**DOI:** 10.1111/eva.70221

**Published:** 2026-03-19

**Authors:** Benjamin Griffin, Riya Gosrani, Jessica Eccles

**Affiliations:** ^1^ Department of Clinical Neuroscience, Brighton and Sussex Medical School University of Sussex Falmer UK; ^2^ University College London Hospital London UK

**Keywords:** ADHD, anxiety disorders, autism: major depressive disorder, evolutionary psychiatry, neurodiversity movement

## Abstract

Mainstream psychiatry continues to interpret neurodivergence through a disease paradigm, assuming that all cases of autism and ADHD reflect disordered brain development. This framing has contributed to the view that elevated rates of co‐occurring psychiatric diagnoses found in neurodivergent populations can be explained through shared mechanisms of neurobiological dysfunction. The neurodiversity movement has challenged this view, reframing neurodiversity as natural variation within human cognition, and emphasizing that much of the associated distress in neurodivergent individuals arises from systemic social barriers, rather than internal dysfunction. In contrast to the disease paradigm's individualizing focus, this relational perspective suggests psychopathology in neurodivergent individuals arises primarily from the poor fit between their cognitive profiles and modern environments. Evolutionary psychiatry may offer a scientific foundation for this reframing. Here, we synthesize evolutionary insights on autism, ADHD, and affective disorders to provide a novel explanation for elevated rates of major depression and anxiety disorders in neurodivergent populations, based upon the principles of evolutionary trade‐offs and mismatch. This perspective offers a scientifically grounded and ethically progressive framework for understanding neurodivergence and its psychiatric comorbidities; one that emphasizes prevention and environmental accommodation, instead of pathologisation and deficit‐correction.

## 1 Introduction

*Neurodiversity* refers to the natural neurocognitive variation found across human populations, including differences in information processing, social cognition, and behavioral regulation (Kapp 2020). Within this framework, *neurotypicality* denotes patterns of neurocognitive development that are statistically most common in the population, while *neurodivergence* refers to *neurodivergent* individuals whose cognitive traits fall outside these common ranges (ibid). The current mainstream approach to understanding neurodiversity within psychiatry is the disease paradigm. Both the ICD‐11 and DSM‐5 categorize deviations from neurotypicality which cause difficulties in meeting societal demands as “neurodevelopmental disorders” (Box 1). Cognitive and behavioral differences in neurodivergent individuals are thus understood to be symptoms of disordered brain development. Epidemiological research robustly associating autism, attention deficit hyperactivity disorder (ADHD), and related forms of neurodivergence such as dyslexia with increased rates of other psychiatric diagnoses across the lifespan has been interpreted as supportive evidence for this framing (Katzman et al. 2017; Hendren et al. 2018; Hossain et al. 2020). Vastly higher rates of major depressive disorder and anxiety disorders particularly are amongst the most consistent findings: in clinical populations, lifetime prevalence rates amongst neurodivergent individuals have been reported to exceed 50% (Torgersen et al. 2006; Joshi et al. 2013).

From the starting point that neurodivergence and other psychiatric diagnoses are forms of disordered brain functioning which are observed to commonly occur together, researchers have hypothesized that shared pathological mechanisms will explain this association. A recent review article entitled ‘Neurobiological Relationships Between Neurodevelopmental Disorders and Mood Disorders’ typifies the logic of this approach: “Neurodevelopmental disorders and mood disorders are not distinct categories but share complex neurobiological foundations. Alterations in mechanisms such as synaptic plasticity, neurotransmitter metabolism, and neural connectivity contribute to developing symptoms in both conditions” (Bertollo et al. 2025, pp. 3).

Findings from psychiatric genomics are used to buttress this framing, with genetic correlations between polygenic scores for autism, ADHD, and other psychiatric diagnoses often taken as evidence of “comorbidity” being genetically determined. A study by Riglin et al. (2020) exploring causal explanations for the association of ADHD and major depressive disorder exemplifies this in its reporting of a Mendelian randomization analysis. After finding that genetic liability to ADHD was associated with increased risk of major depressive disorder, the authors conclude that their results establish a deterministic “causal effect of ADHD genetic liability on subsequent major depression” (pp. 1) delivering the pessimistic verdict that “effective treatment of ADHD… is unlikely to consistently prevent the development of depression” (pp. 7).

Despite the confidence of these conclusions, the inferences drawn rest upon the assumptions of the biomedical paradigm. Mendelian randomisation analysis cannot, in fact, distinguish between direct causal biological pathways and indirect, environmentally mediated ones (Davies et al. 2019). While Riglin et al. (2020) adjust for possible environmental confounders, these are limited to a narrow set of variables: sex, early social adversity (operationalized as parent home ownership and crowding during pregnancy only), maternal education, and maternal prenatal depression. These arguably do not capture the full spectrum of educational, occupational, interpersonal, and structural challenges that neurodivergent individuals are increasingly well appreciated to face across their lives (Singer 2017). The authors' interpretation—that ADHD genes directly causally predispose to major depressive disorder—is therefore not a neutral reading of the data, but one shaped by a prior assumption that ADHD represents defective biology. This assumption renders it intuitively plausible that the “disease‐causing genes” of ADHD also give rise to further disorders. In doing so, the study exemplifies a broader tendency found across some biomedical psychiatric research: to interpret concomitance as further proof of the pathological nature of neurodivergence.

In recent years, however, challenges to this framing have emerged. Evolutionary psychiatry is a burgeoning field that seeks to extend explanations for psychiatric diagnoses beyond proximate mechanistic accounts, addressing the apparent evolutionary paradox of their high prevalence in modern populations, despite natural selection's tendency to remove maladaptive traits (Keller and Miller 2006; Abed and John‐Smith 2022). In doing so, evolutionary psychiatry draws on the classic distinction recognized within biology between *proximate explanations*, which describe *how* traits and disorders arise through immediate biological mechanisms, and *ultimate* or *evolutionary explanations*, which address *why* such traits persist in populations over evolutionary time. In answering this question, evolutionary psychiatry has introduced adaptationist perspectives on neurodivergence, which have cast doubt on whether all cases of autism and ADHD should be understood as constituting disorders (Swanepoel 2024).

This adaptationist perspective overlaps in important ways with the goals of the neurodiversity movement; a social justice initiative which emphasizes that many of the struggles faced by neurodivergent individuals arise from systemic barriers rather than intrinsic deficits, and advocates for changes in the language used to describe neurodivergent traits, seeking to replace deficit‐focused terminology with affirming, descriptive alternatives (Hunt and Procyshyn 2024; Shaw et al. 2024). In this article, we bring theoretical insights from evolutionary psychiatry to bear on the question of why neurodivergence is so often concomitant with other psychiatric diagnoses, particularly major depressive disorder and anxiety disorders. We aim to show how an evolutionary perspective not only exposes the hidden assumptions underlying reductive biomedical interpretations, but also offers a more coherent and humane account of why neurodivergent individuals are at increased risk of psychopathology in modern societies, which dovetails with key principles of the neurodiversity movement.

BOX 1 **On imperfect terminology.**

Language is not neutral. The neurodiversity movement has highlighted how biomedical terminology often carries ableist assumptions, framing neurocognitive differences as deficits. This reflects both the political and conceptual importance of language in reframing neurodivergence. While many autistic self‐advocates and scholars have advanced more neutral alternatives—such as identity‐first language (autistic people) and terms like autistic spectrum or autistic spectrum condition (Bottema‐Beutel et al. 2021)—equivalent shifts have lagged for ADHD, leaving no widely accepted non‐pathologising terminology (French et al. 2025). For clarity, we therefore retain the acronym “ADHD” in this manuscript, while rejecting the assumption that such cognitive profiles are invariably disordered.

## 2 Diagnostic Heterogeneity, Evolutionary Trade‐Offs, and Evolutionary Mismatch: Foundational Concepts in Understanding Evolutionary Accounts of Autism and ADHD

It is important at the outset to clarify the scope of the present argument. Autism and ADHD are highly heterogeneous phenomena, encompassing a wide range of cognitive and behavioral profiles; from individuals with average or above‐average intellectual functioning and relatively circumscribed difficulties to rarer cases involving profound developmental impairment and lifelong dependence on care. A minority of presentations arise from rare genetic syndromes or severe environmental insults—for example, Phelan‐McDermid Syndrome or foetal valproate exposure respectively—which can produce globally impairing neurodevelopmental outcomes. The evolutionary considerations advanced in this paper are not intended to apply to such cases, but rather to the more common highly heritable forms of autism and ADHD that present as stable, trait‐like variation in cognition and behavior.

Indeed, several features of these more common presentations make them especially amenable to adaptive evolutionary explanation. These presentations of autism and ADHD are massively polygenic and highly heritable, with twin studies consistently estimating heritability at around 80% (Sandin et al. 2017; Grimm et al. 2020). They also emerge early in development and are relatively prevalent in the population, with estimated rates of approximately 5%–8% for ADHD (Song et al. 2021; Ayano et al. 2023; Salari et al. 2023) and 0.6%–3% for autism (Global Burden of Disease Study 2021 Autism Spectrum Collaborators 2025; Shaw et al. 2025) (Box 2). These characteristics make these presentations of autism and ADHD highly visible to natural selection. As such, evolutionary psychiatrists have argued that if autism and ADHD were purely deleterious, evolutionary theory would predict that contributory alleles would be far rarer in human populations (Swanepoel 2024). The persistence and prevalence of these traits have therefore motivated the development of adaptationist accounts that challenge their classification within mainstream biomedical psychiatry as straightforward instances of disorder.

It should also be highlighted that such adaptive accounts are fully compatible with extreme expressions of these traits being harmful or impairing. Far from being unique to autism or ADHD, this reflects a general principle in evolutionary biology: traits can be maintained by selection despite the presence of costly or maladaptive expressions at the extremes of their distribution (Abed and John‐Smith 2022). The concept of an autistic spectrum is naturally aligned with this distributional view.

BOX 2 **Uncertainties in prevalence rates.**

Prevalence estimates of both autism and ADHD vary substantially across studies, reflecting multiple sources of heterogeneity, including evolving diagnostic criteria and thresholds, differences in clinical and research practices, study design, and case ascertainment. Additional contributing factors include cultural variation in symptom recognition, health system infrastructure and access, use of screening versus formal diagnosis, age and cohort effects, comorbidity and diagnostic overshadowing, and geographical and socio‐economic differences. More broadly, the marked global increase in mental health problems among children and adolescents observed since the COVID‐19 pandemic underscores the importance of environmental and contextual influences on psychiatric prevalence (Ng and Ng 2022). The rapidity of these changes cannot plausibly be explained by genetic factors alone, underscoring the sensitivity of mental health outcomes to environmental conditions.

With this scope clarified, two key core concepts from evolutionary medicine are integral to understanding adaptationist explanations of neurodivergence:

- Evolutionary trade‐offs.
- Evolutionary mismatch.

### 2.1 Evolutionary Trade‐Offs

A common but misguided notion is that evolution has selected for a single ‘optimal’ type of mind. In reality, natural selection operates through compromises rather than perfection, with evolutionary trade‐offs being ubiquitous in living organisms (Garland et al. 2022) (Table 1). The enhancement of one evolved characteristic, or trait, often comes at the expense of another, and what counts as adaptive is contingent on the environment; no phenotype can be ideal across all contexts, and environments are inherently unpredictable and changeable. As such, all minds are inherently suboptimal in some respects, and every cognitive profile entails both strengths and vulnerabilities.

**TABLE 1:** Non‐mutually exclusive types of evolutionary trade‐off; adapted from Garland et al. (2022).

Type	Description	Example
Allocation constraints	Limitations in the distribution of finite resources (e.g., energy, nutrients) among competing biological functions necessitates compromises	Limited resources force plants to trade‐off between producing many small seeds or fewer larger seeds with greater individual survival prospects
Functional conflicts	Enhancing one trait often comes at the cost of impairing another	Harder, more mineralized bones are stronger under compression but less flexible, making them more brittle and prone to fracture
Shared biochemical pathways	Situations where multiple physiological functions rely on the same biochemical resources creates trade‐offs as enhancing one function may limit the resources available for others, leading to functional conflicts	Steroid hormones derived from cholesterol regulate both stress responses and reproduction, such that elevated stress hormone production can suppress reproductive function
Antagonistic pleiotropy	When a single gene affects multiple traits, enhancing one trait's fitness may come at the expense of reducing another's	There is growing evidence to suggest the ApoE4 allele may have been maintained by selection due to early‐life reproductive advantages, despite increasing susceptibility to late‐life neurodegenerative and cardiovascular disease
Ecological circumstances	Trade‐offs that arise because a trait beneficial in one ecological or environmental context may be disadvantageous in another	High melanin levels protect against UV radiation in equatorial regions but increase vitamin D deficiency risk at higher latitudes
Sexual selection	Trade‐offs between traits that increase mating success and those that enhance survival or other aspects of fitness	The peacock's ornate tail boosts mating success while reducing survival due to energetic and predation costs

Because a single ‘best’ phenotype is unattainable, evolution has plausibly favoured cognitive diversity rather than uniformity (Moran 1992; Bergmüller and Taborsky 2010; Montiglio et al. 2013). Variation in cognitive and behavioral traits has been proposed to be maintained through mechanisms such as social niche specialization, balancing selection, negative frequency‐dependence, and adaptive developmental plasticity (Hunt and Jaeggi 2022) (Table 2). These processes allow different cognitive styles to persist across generations by conferring context‐dependent advantages in different environments. Individual differences—including those characteristic of neurodivergence—can thus be understood as products of these evolutionary dynamics rather than aberrations from some singular normal point of optimality.

**TABLE 2:** Evolutionary mechanisms by which trait diversity is maintained within species; adapted from Hunt and Jaeggi (2022).

Type	Description	Example
Social niche specialization	The development of distinct traits within a species that allow individuals to occupy complementary social roles, minimizing competition and supporting group function	In many social insect species, individuals specialize into roles such as foraging or defense, reducing competition and increasing colony efficiency
Balancing selection	The process by which multiple alleles are maintained in a population because they confer advantages under different conditions	The sickle‐cell allele has been maintained in malaria‐exposed populations because it confers resistance, despite causing disease in homozygous individuals; this is termed heterozygote advantage
Negative frequency‐dependence	The fitness of a trait increases as it becomes rarer in the population, typically stabilizing at low frequency in the population	In Batesian mimicry, harmless species benefit when they resemble toxic models while rare, but lose this advantage as they become common and predators learn to distinguish them
Adaptive developmental plasticity	The capacity of an organism to adjust its development in response to environmental cues, producing phenotypic variations that enhance fitness in specific contexts	In many species of frog and toad, genetically identical tadpoles will develop different body shapes depending on whether or not they are exposed to predator chemical cues during development

**FIGURE 1:**
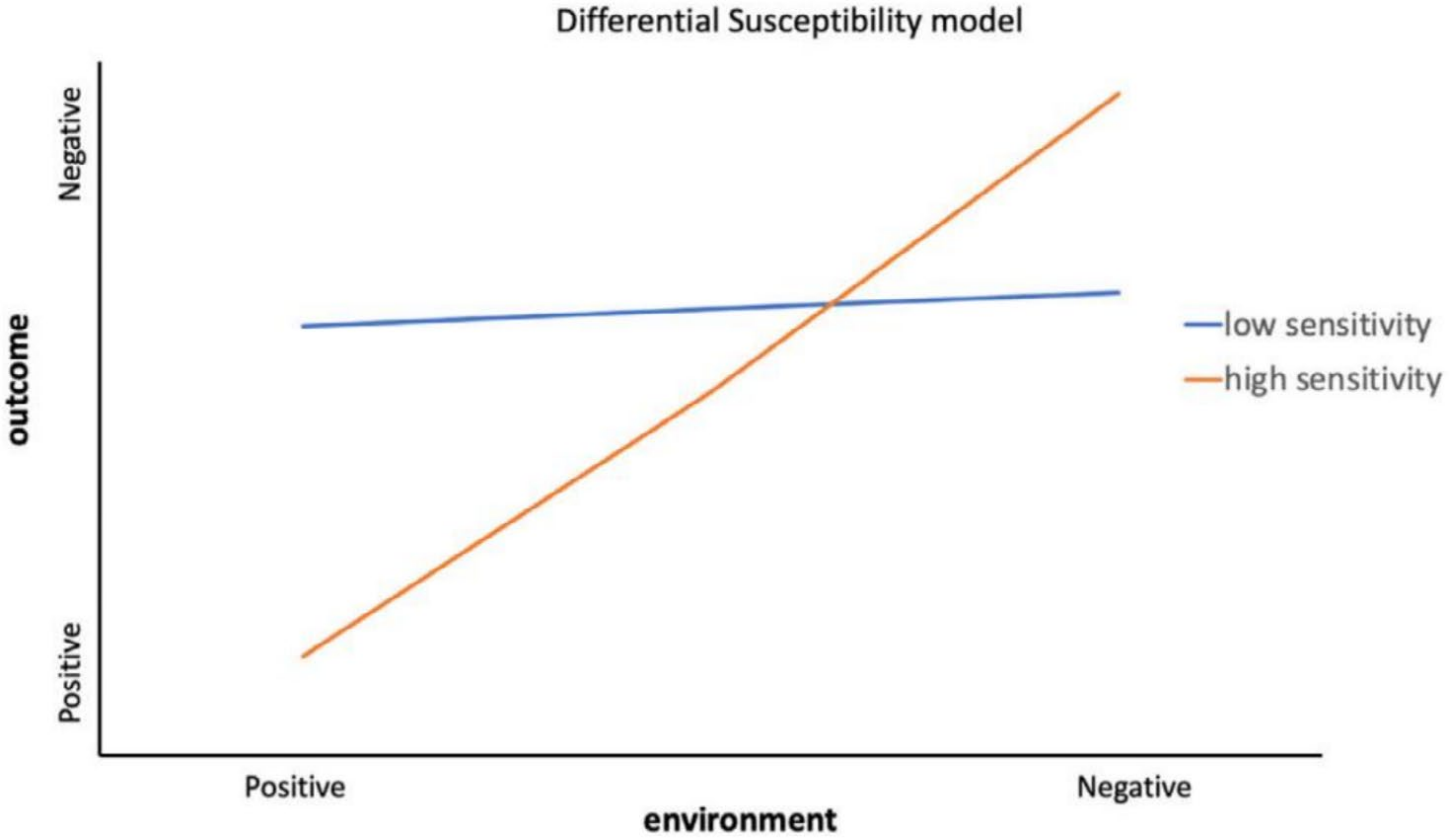
Differential susceptibility illustrated (Assary 2023).

Differential susceptibility is a related concept, denoting individual variability in responsiveness to environmental influences (Belsky and Pluess 2009; Ellis et al. 2011; Assary 2023) (Figure 1). The ‘orchids versus dandelions’ heuristic illustrates this variation (Swanepoel 2024): more environmentally sensitive individuals (orchids) trade off experiencing more negative outcomes under adverse conditions against deriving greater benefits from supportive environments, while less environmentally sensitive individuals (dandelions) trade off greater resilience across contexts against being less able to thrive in specific niches. ‘Orchids’ have ‘spiky’ cognitive profiles, with uneven distributions of cognitive strengths and weaknesses, rather than the more uniform abilities found in ‘dandelions’ (Pluess 2015; Wilson 2024). Traits associated with neurodivergence, such as those seen in autistic or ADHD individuals, can be conceptualized as orchid‐like: highly sensitive to environmental conditions, which may amplify vulnerability in unsupportive contexts but confer exceptional capacities in specific well‐suited environmental niches. Recent evidence further suggests that autism diagnosed later in life is genetically distinct from early‐diagnosed autism and shows stronger polygenic overlap with mental health conditions (Zhang et al. 2025). This pattern is consistent with the possibility that some environmentally sensitive (“orchid‐like”) cognitive profiles may remain unrecognized in early development, but become increasingly vulnerable to depression and anxiety as social demands intensify and environmental support fails to keep pace.

From this perspective, the facts that traits associated with autism, ADHD, and other forms of neurodivergence deviate from species‐typical statistical norms, and that individuals expressing these traits may exhibit relative difficulties in some cognitive or behavioral domains compared to neurotypical individuals, do not necessarily imply they constitute disorders. Rather—as will be expanded upon—being neurodivergent likely confers distinctive adaptive advantages in specific ecological or social niches. This evolutionary perspective aligns closely with the theoretical foundations of the neurodiversity movement. As Singer (2017) articulated in the seminal text ‘Neurodiversity—the birth of an idea’, “just as biodiversity is essential to ecosystem stability, so neurodiversity may be essential for cultural stability” (pp. 21), providing “a repository of types who may come into their own under unforeseeable circumstances” (pp. 87).

### 2.2 Evolutionary Mismatch

**FIGURE 2:**
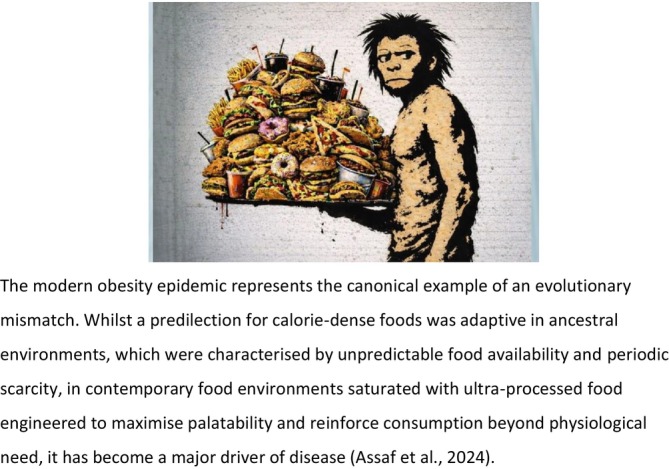
Evolutionary mismatch exemplified.

Biological evolution proceeds through random genetic variation and natural selection acting over many generations, rendering it a relatively slow and gradual process. By contrast, environments can change rapidly. In humans, this disparity has been amplified by the uniquely human process of cumulative cultural evolution; the capacity to accumulate, refine, and transmit knowledge, technologies, and social practices across generations. Through cumulative culture, humans have transformed their social and ecological environments at an accelerating rate, far exceeding that of biological evolution. Consequently, many psychological and physiological traits that evolved to meet adaptive challenges in ancestral environments are now expressed in radically altered contexts, creating evolutionary mismatches between evolved dispositions and contemporary demands (Griffin et al. 2025) (Figure 2). Importantly, the fact that certain traits are associated with harm in modern settings does not imply that they constitute failures of function; rather, they may once have conferred significant advantages under the ecological and social conditions in which they evolved.

Although contemporary hunter‐gatherer societies are not exact replicas of our ancestral environments, anthropological studies of such groups nonetheless provide valuable, if imperfect, windows into the conditions under which human cognition and behavior evolved (Chaudhary and Swanepoel 2023). Such research has strongly suggested that modern lives—especially within Western, Educated, Industrialised, Rich, and Democratic (WEIRD) societies (Henrich 2021)—differ profoundly from those of ancestral hunter‐gatherer groups. Whereas ancestral humans lived in small, kin‐based communities characterized by such features as strong social interdependence, egalitarianism, and continuous physical activity, WEIRD environments are defined by large, impersonal social networks, rigid institutional hierarchies, sedentary lifestyles, and increasingly highly specialized forms of cognitive labor.

Differences in childhood environments may be particularly important for understanding the concomitance of neurodiversity and other psychiatric diagnoses (Chaudhary and Swanepoel 2023). Children today grow up in nuclear families rather than multi‐age, alloparental communities; they engage in structured, sedentary schooling rather than exploratory, play‐based learning; and they experience reduced autonomy and physical activity. These differences may be particularly relevant for understanding the concomitance of neurodivergence and other psychiatric diagnoses. Environments that demand uniformity in attention, activity, and social learning styles are likely to generate heightened stress and exacerbate vulnerabilities in individuals whose cognitive profiles diverge most from these modern expectations.

Taken together, the principles of evolutionary trade‐offs and evolutionary mismatch provide a foundation for understanding adaptive explanations for neurodivergence. Indeed, most evolutionary theories present autism and ADHD as specialized neurocognitive profiles characterized by trade‐offs that are particularly mismatched to modern environmental conditions.

## 3 Adaptationist Accounts of Autism

Autism has been interpreted through a range of evolutionary frameworks that seek to explain the persistence and distribution of autistic traits in human populations. These include Crespi's (2016) ‘high intelligence imbalance’ hypothesis and Del Giudice's (2018) slow‐life history model (Table 3), as well as Baron‐Cohen's (2009) empathisising‐systemising hypothesis. A recent comprehensive investigation by Hunt and Jaeggi (2025), which systemically reviewed evidence from multiple empirical domains and evaluated competing models against formal explanatory criteria, concluded that the empathising–systemising hypothesis is superior in explanatory scope and empirical sufficiency.

**TABLE 3:** Two alternative evolutionary hypotheses of autism.

Crespi (2016) high intelligence imbalance hypothesis	Proposes that autistic traits arise from an uneven amplification of specific components of intelligence rather than from globally reduced cognitive ability. Although autism is often associated with below‐average IQ at the phenotypic level, genetic studies show substantial overlap between alleles linked to autism risk and those associated with high intelligence; Crespi argues that this apparent paradox can be resolved if autism commonly reflects enhanced but imbalanced cognitive capacities, particularly in analytical or mechanistic domains, alongside relative weaknesses in integrative or social processing
Del Giudice's (2018) slow‐life history model	Situates autistic traits within life history theory, which examines how organisms allocate limited resources between growth, maintenance, reproduction, and social investment. Within this framework, autism‐related traits are associated with a slower developmental strategy. These are characterized by long‐term planning, high behavioral control, reduced social signaling, and lower orientation toward short‐term interpersonal competition, representing one end of continuous variation in human developmental strategies. This has been proposed to represent a mating strategy which, in non‐pathological expression, is associated with lower interest in short‐term mating and greater emphasis on partner‐specific investment and long‐term relational commitments (Del Giudice et al. 2010)

The empathising‐systemising hypothesis proposes that autistic cognition reflects a heightened capacity for systemising—the drive to analyse, understand, and manipulate rule‐based, technical, or physical systems—traded off against a relative reduction in empathising, or intuitive understanding of other minds. In The Pattern Seekers, Baron‐Cohen (2020) further situates systemising within a deep evolutionary narrative, proposing that the evolution of this cognitive mechanism underlies human inventiveness, potentiating exponentially increased rates of cumulative cultural evolution.

Indeed, the empathising–systemising hypothesis is supported by converging evidence from multiple domains (reviewed in Baron‐Cohen 2020). Behaviorally, autistic individuals reliably score higher on systemising measures than neurotypical controls, and autistic traits are overrepresented in STEM disciplines and occupations that require advanced systemising abilities. Additional support comes from the broader autism phenotype: subclinical expression of autistic traits in first‐degree relatives of autistic individuals. Parents of autistic children frequently perform better on systemising tasks and are more likely to work in technical fields such as engineering, consistent with a heritable cognitive style potentially maintained through evolutionary processes such as social niche specialization.

**FIGURE 3:**
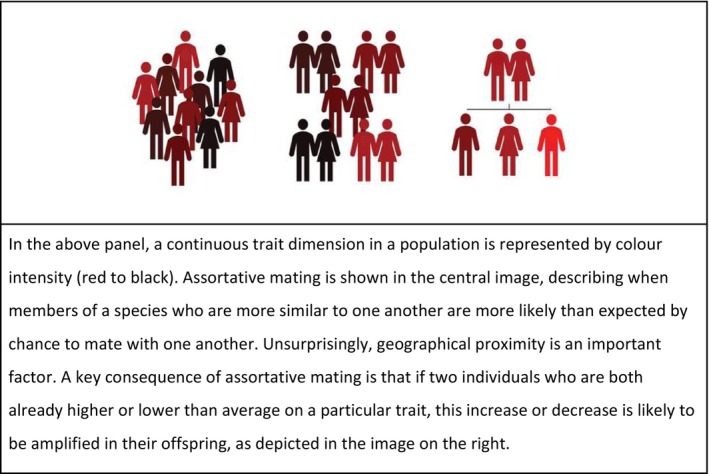
Assortative mating (Versluys et al. 2021).

This theory has been particularly influential not only because it integrates multiple empirical findings but also because it has generated novel, falsifiable predictions. One illustrative example is a study combining the empathising‐systemising hypothesis with the evolutionary principle of assortative mating (Figure 3) to predict higher autism prevalence in regions with a greater concentration of systemisers (Roelfsema et al. 2012). Consistent with the prediction, the prevalence of autism in Eindhoven—a major technological hub—was 2.3%, compared to 0.8% and 0.6% in Haarlem and Utrecht, respectively. This finding provides an intriguing perspective on the rising prevalence of autism, suggesting they may not just reflect changing diagnostic practices alone.

From an adaptationist perspective, the cognitive profile characteristic of autistic individuals may thus reflect an evolutionary maintained specialisation advantageous in particular ecological and social contexts. Reser (2011) has speculated that, in ancestral settings, reduced social motivation with increased preference for solitary activity may have conferred adaptive advantages by minimising exposure to costly social conflicts. In small‐scale societies, status competition, coalitionary dynamics, and interpersonal disputes are proposed to have risked incurring significant energetic and reputational costs. On this view, individuals more oriented toward solitary, technical, or material problem‐solving—such as tool‐making, tracking, or resource extraction—may therefore have avoided these social risks while contributing uniquely valuable skills to the group.

However, this same profile may be especially vulnerable to evolutionary mismatch in modern environments. WEIRD societies are characterized by unprecedented levels of social complexity, rapid cultural and technological change, and highly structured institutional environments. Many of these features contrast sharply with the small, kin‐based, and relatively stable social groups in which human cognitive capacities evolved. Educational and occupational systems in many WEIRD societies are also structured around prolonged formal schooling, high levels of social coordination, and increasingly abstract and unpredictable social demands, which may further exacerbate challenges for those whose cognitive strengths lie in systematic, rule‐based domains rather than fluid social navigation. The demands of navigating large, impersonal social networks and engaging in frequent interactions with unfamiliar individuals place a premium on rapid, flexible social inference and implicit perspective‐taking; domains in which autistic individuals often experience relative difficulties. Additionally, modern environments often reward constant multi‐tasking, rapid task‐switching, and tolerance of high sensory load, creating further disadvantage for the cognitive and sensory profiles characteristic of autistic minds.

## 4 Adaptationist Accounts of ADHD

Adaptationist accounts of ADHD move beyond deficit‐based psychiatric models by highlighting the potential adaptive advantages of the traits involved (Williams and Taylor 2006). This includes moving away from focusing upon core ‘symptoms’ of inattention, hyperactivity and impulsivity—which frame the condition as impaired functioning—to that of being adventurous, more creative, and energetic. Evolutionary perspective on ADHD may thus provide a possible repository for more affirmative diagnostic perspective and terminology, which are currently lacking (French et al. 2025). These adaptationist approaches primarily draw on evolutionary perspectives that include trade‐offs and mismatch, as well as life history theory.

Individuals are faced with countless decisions each day, across a wide range of environments. To navigate these effectively, they must skillfully balance exploiting known, reliable options with exploring uncertain alternatives; a delicate equilibrium termed the exploration‐exploitation trade‐off (Jami et al. 2025). This balance underpins adaptive decision‐making and reward‐seeking behaviour. In individuals with ADHD, this equilibrium is often relatively skewed towards tending to favour exploration over exploitation. This reflects heightened reward‐seeking tendencies, which are often seen in modern‐day society as difficulties in maintaining stable, goal‐directed choices.

An illustrative example of this context‐dependent advantage comes from research among the Ariaal people of Kenya. A study found that men carrying a novelty‐seeking associated variant of the DRD4 gene were better nourished and healthier when living a nomadic, mobile lifestyle, but exhibited poorer outcomes when living in settled, agricultural communities (Eisenberg et al. 2007). This demonstrates how ADHD traits which are associated with heightened exploration and impulsivity may once have been adaptive, enhancing survival in dynamic or unpredictable environments (Swanepoel et al. 2017). Mismatch theory builds on this idea by situating these traits within modern environments.

Swanepoel et al. (2017) highlight that most ADHD diagnoses to date have primarily been made within the context of schooling, suggesting that in the highly structured and rigid environment of schools, previously advantageous traits—such as a tendency to explore, seek novelty, and challenge established norms—are often reprimanded or pathologised, rather than recognized as potential strengths. Even with the recent rise in adult ADHD diagnoses, mismatch theory remains relevant, as both modern‐day schools and work environments—particularly sedentary, desk‐based jobs—differ substantially from the ancestral environments in which these traits developed. These novel environments often require sustained attention and limited scope for immediate rewards, making it difficult for individuals with ADHD to remain engaged.

While ADHD is often characterized by difficulties sustaining attention, this overlooks the increasingly recognized phenomena of hyperfocus, which is described as periods of intense, sustained concentration on activities that are intrinsically interesting or rewarding (Oroian et al. 2025). ADHD may be better understood as a variation in attention control and responsiveness to reward, rather than a mere lack of attention. Dopamine pathways involved in motivation and reward processing are thought to play a key role in this dynamic (Volkow et al. 2009). From an adaptationist perspective, the ability to hyperfocus on highly stimulating or novel tasks could have been advantageous in ancestral environments, facilitating rapid learning, problem‐solving, or persistence under time pressure. However, in modern sedentary settings that prioritize prolonged, externally directed focus on often monotonous tasks, this same attentional pattern may be pathologised. Hyperfocus thus highlights a broader point of mismatch: ADHD traits may not signify impaired functioning universally, but rather reflect an attentional style that is more attuned to variable, high‐stimulation environments than to the uniform demands of contemporary educational and occupational systems.

This perspective raises important ethical questions about the appropriateness of prescribing stimulant medications to those with ADHD, particularly children (Swanepoel 2021). While medication can improve the ‘goodness of fit’ between a child's cognitive profile and their environment, potentially reducing distress and enhancing functional outcomes, such evolutionary perspectives caution against assuming pharmacological intervention as the default solution, arguing medications should not be construed as treating symptoms of a disorder. If many ADHD traits primarily reflect mismatch with school or modern work environments, it may be equally or more important to adapt these contexts. Evolutionary perspectives thus encourages judicious prescribing that considers both individual benefits and the potential for systemic or environmental adaptations to reduce reliance on medication. For example, research indicates that engagement in physical activity and structured movement‐based settings can be associated with improved attention, self‐regulation, and overall well‐being (Sun et al. 2022).

Building on these ideas, Le Cunff (2024) applies evolutionary theory to argue that working with, rather than against, natural tendencies in ADHD may lead to better outcomes. Drawing on the concepts of evolutionary trade‐offs and mismatch, she reframes traits such as impulsivity and reward‐seeking as ‘hypercuriosity’, drawing a parallel to the captivity effect in non‐human primates, where animals in low‐risk, captive environments display heightened curiosity (Forss and Willems 2022). The captivity effect is thought to be due to increased free time and reduced risk exposures in confined settings. Modern‐day environments, with their structured and low‐risk demands, may similarly amplify hypercurious behaviors in individuals with ADHD. To support and direct hypercuriosity positively, approaches like personalized learning, game‐based activities, Montessori classrooms, and outdoor activities may enhance natural curiosity and adaptive functioning (Le Cunff 2024).

Trade‐off and mismatch perspectives have helped explain why ADHD traits may manifest differently depending on environmental context. Life History Theory augments this by accounting for why certain groups or populations may exhibit higher prevalence of ADHD‐related traits compared to others. Life History Theory provides a framework for understanding how energy is allocated across growth, survival, and reproduction, with trade‐offs shaped by environmental conditions (Sear 2020). High perceived extrinsic mortality, particularly in low‐SES contexts, signals a greater likelihood of dying before reproducing, incentivizing early reproductive effort and risk‐taking behaviors. ADHD has been widely linked to early environmental risk factors such as low parental education, low socioeconomic status, childhood maltreatment, and adversity. Therefore, children who experience unstable or abusive environments may adopt a ‘fast’ life‐history strategy, prioritizing immediate rewards and early reproduction over long‐term health and planning. This helps explain why the constellation of risky behaviors often observed in individuals with ADHD may, from an evolutionary perspective, confer adaptive advantages.

## 5 Contextualising Major Depressive Disorders and Anxiety Disorders in Neurodivergent Populations

A key insight from evolutionary psychiatry is that low mood and anxiety are evolved defensive systems, shaped to protect individuals from threats and facilitate adaptive functioning in potentially hostile environments (Nesse 2018). Low mood has been hypothesized to plausibly conserve energy, promote reflection, and signal disengagement from unattainable goals (Nesse 2000; Andrews and Thomson 2009), while anxiety heightens vigilance and motivates avoidance of potential dangers (Bateson et al. 2011). A central evolutionary explanation for vulnerability to mood and anxiety disorders is that these defensive responses are inherently prone to excess expression and dysregulation: because the costs of failing to respond to genuine threats historically outweighed the costs of false alarms, natural selection favoured systems that are easily activated, even at the risk of overreacting (Nesse 2023). Major depressive disorder and anxiety disorders are thus postulated to arise when these defensive responses become chronically overactivated or misaligned with environmental demands, producing sustained and disproportionate responses.

This model of anxiety and low mood as evolved defenses is often formalized through Nesse's smoke detector principle, which highlights the asymmetric costs of missed versus false alarms: because failing to respond to a real threat can be catastrophic, natural selection favours low activation thresholds, making defensive responses easy to trigger (Nesse 2005; Nesse 2018). While likely adaptive in ancestral contexts, these low thresholds become harmful when chronic, diffuse, or evolutionarily novel stressors continually activate defense systems.

**FIGURE 4:**
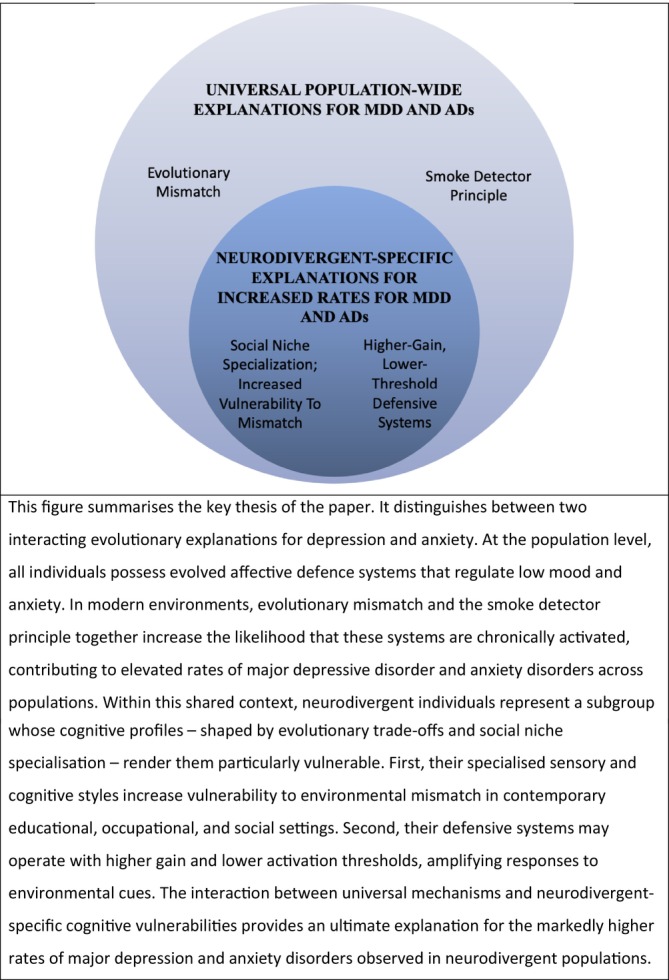
Universal population‐wide and neurodivergent‐specific mechanisms for major depressive disoder and anxiety disorders.

Evolutionary mismatch is central to this dynamic. Modern environments—characterized by large impersonal networks, rigid institutional demands, constant novelty, and sustained sensory input—are poorly calibrated to these ancestral expectations, and as such plausibly can act as persistent low‐grade triggers for these defensive systems, raising baseline arousal and increasing vulnerability to affective dysregulation. This framework has been proposed to account for the high prevalence of depression and anxiety in contemporary societies, including the rise in affective symptoms observed particularly among young people in recent decades. In this sense, evolutionary mismatch provides a ‘universal’ population‐level explanation for increased susceptibility to mood and anxiety disorders across populations (Figure 4).

However, for neurodivergent individuals, this vulnerability is heightened. As described previously, their cognitive and sensory profiles often entail evolutionary trade‐offs that may have conferred advantages in ancestral social niches but particularly increase their susceptibility to being significantly mismatched to aspects of modern environments. To the extent that these cognitive profiles reflect more specialized ways of processing information, attention, or sensory input, they may be especially sensitive to environments that are inflexible, overstimulating, or structured around neurotypical expectations, placing neurodivergent individuals at greater risk of pronounced evolutionary mismatch.

A second neurodivergent‐specific explanation may exist. Using the signal‐detection framework (Nesse 2005), their defensive systems may also operate with lower thresholds and higher gain, making them more likely to detect and respond strongly to environmental cues than neurotypical individuals. Whereas mismatch concerns the frequency and persistence of stressors in the environment, this mechanism concerns sensitivity to those stressors: heightened perceptual acuity, pattern detection, or intolerance of uncertainty may increase the likelihood that ambiguous or low‐level cues are interpreted as salient or threatening. In such contexts, greater activation of defensive systems is not only plausible but expected, offering a coherent explanation for the consistently higher rates of major depressive disorder and anxiety disorders among neurodivergent populations.

This evolutionary reframing departs from deterministic biomedical narratives, which often construe major depressive disorder and anxiety disorders among neurodivergent individuals as inevitable consequences of their “disordered” brains. Instead, these outcomes are situated within the interaction between evolved defense systems and modern environments, shifting the explanatory focus from individual deficits to a poor fit with current environmental conditions. This perspective is inherently relational in its ontology: it understands psychological suffering not as a property of isolated brains, but as emerging from the dynamic relationship between individual cognitive dispositions and their ecological, social, and cultural contexts. By foregrounding this relational interplay, it highlights how vulnerability and resilience are context‐dependent rather than intrinsic qualities. Critically, if much of the elevated risk is socially mediated, it is also potentially preventable.

This underscores the need for systemic interventions that reduce the chronic activation of these defensive systems in neurodivergent individuals. These may include, but are not limited to: neurodiversity‐affirming education and workplaces (Davies et al. 2023); sensory‐accessible environments (Malcolm 2022); flexible institutional structures (Frolli et al. 2023); supportive communities (Tobin et al. 2014); and sustained efforts to dismantle stigma and discrimination (Turnock et al. 2022). Rather than accepting elevated rates of major depressive disorder and anxiety disorders as inevitable “comorbidities,” this perspective reframes them as structurally mediated outcomes of environmental mismatch, which critically are amenable to prevention. This has important political and ethical implications. The prevailing biomedical framing of neurodivergence—as disordered brains that naturally give rise to further psychiatric “pathology”—implicitly naturalises and depoliticizes this suffering. It shifts responsibility onto individuals and their biology, rather than onto the social systems that generate chronic stress and poor mental health. Furthermore, if neurodivergent cognitive profiles reflect the expression of evolved neurobiological systems rather than intrinsic defects, then attempts at “cure” are unlikely to be feasible or desirable, and continued investment in pathology‐focused models risks diverting resources away from more effective structural and environmental interventions.

Indeed, an evolutionary perspective highlights that these psychiatric outcomes are neither inevitable nor immutable. Increased rates of major depressive disorder and anxiety disorders in neurodivergent populations are best understood not as further proof of defective mental functioning, but as the predictable consequence of evolved neurodivergent cognitive profiles operating in a society to which they are often profoundly mismatched. Reframing these phenomena in this way aligns closely with the goals of the neurodiversity movement, which recognizes that much of what is pathologised in neurodivergent lives is socially produced rather than biologically predetermined. A society that genuinely values and accommodates cognitive diversity would not only reduce psychiatric burden but would transform what is currently framed as disorder into a recognized form of natural mental variation; variation that may have historically contributed to group‐level adaptability and cultural innovation, supporting the evolutionary success of our species.

## 6 Novel Predictions

Evolutionary approaches in biology, particularly as have been applied to understanding human psychology, have long been accused of constituting no more than pseudoscientific “just‐so” stories (Pigliucci and Kaplan 2000; Smith 2020; Maung 2025). The philosophical ammunition for these critiques has largely been derived from Popper's widely influential claim that scientific theories can be demarcated from other forms of knowledge by the falsifiability criterion (Popper 1963) (Box 3). However, such sweeping characterizations are untenable.

BOX 3 **Popperian falsification.**

The most fundamental question in the philosophy of science is the *demarcation problem*: how to distinguish, or demarcate, scientific theories from non‐scientific or pseudoscientific ones. Karl Popper's widely influential answer was *falsificationism*; the view that scientific theories must generate testable predictions that could, in principle, be shown to be false (Popper 1963). Although Popper's solution to the demarcation problem is now widely regarded as oversimplistic (Pigliucci and Boudry 2013), the emphasis on generating novel, discriminative, and empirically testable predictions has nevertheless played a central role in scientific progress.

Whilst there are instances of evolutionary psychology and psychiatry hypotheses selectively sampling proximate evidence and failing to stipulate how they could be subjected to falsification via testing, much contemporary evolutionary psychology and evolutionary psychiatry makes systematic use of the hypothetico deductive method, generating discriminative and falsifiable predictions (Griffin et al. 2025). Indeed, bringing the evolutionary perspective to bear on human biology has a significant theoretical strength in its ability to predict phenomena that are completely unexpected from the standpoint of proximate frameworks; a feature Lakatos (1978)—another influential 20th Century philosopher of science—argued to be a particularly important feature of generative, progressive scientific research programs. Furthermore, recent work by Hunt and Jaeggi (2025) provides a rigorous framework through which evidence synthesis and analysis can be conducted, allowing for further sharpening of hypothesising in evolutionary psychology and psychiatry.

With this backdrop in mind, the present account of how neurodivergent cognitive traits interact with evolved affective defense systems and modern environments is intended to be explicitly predictive rather than merely retrospective. Several of the specific predictions outlined below align with and help to integrate a broader growing empirical literature that has documented environmental moderators of mental health outcomes in neurodivergent populations. For example, loneliness has been identified as a significant risk factor for depression and suicidal ideation among autistic individuals, while social support functions as a protective factor (Hedley et al. 2018; Pelton et al. 2020; Quadt et al. 2024). While these findings have not been interpreted in explicitly evolutionary terms to date, they are consistent with the thesis of this paper. Framing these observations within evolutionary psychiatry thus not only unifies disparate findings but also clarifies the conditions under which neurodivergent traits are likely to confer vulnerabilities or advantages.

1. Cross‐Cultural Variability: Populations living in less industrialized or less neurotypical‐structured societies may show lower rates of psychiatric concomitance among neurodivergent individuals, reflecting the interaction between evolved cognitive traits and environmental mismatch rather than inherent pathology.
2. Interventions: Structural interventions—such as flexible schooling, neurodiversity‐affirming workplaces, or sensory‐accessible spaces—will reduce the risk of major depressive disorder and anxiety disorders developing in neurodivergent individuals and produce measurable reductions in depressive and anxiety symptoms.
3. Mismatch‐Dependent Physiological Activation: Physiological markers of stress (e.g., cortisol, heart rate variability) will be more elevated in neurodivergent individuals in environments mismatched to their cognitive profiles, with reductions observed in tailored, supportive settings.
4. Occupational Alignment: Neurodivergent individuals whose occupations leverage their cognitive strengths (e.g., for autistic individuals, tasks requiring intense focus, pattern recognition, and systemising) will show reduced rates of major depressive disorder and anxiety disorders symptoms, as well as higher subjective well‐being, than neurodivergent peers in roles misaligned with their profiles.
5. Polygenic Signal Interaction With Environment: The relationship between polygenic risk for neurodivergence and psychiatric outcomes will be moderated by environmental fit. Outcomes should be most pronounced in contexts lacking accommodations for neurodivergent cognitive styles, whereas risk may appear attenuated where supportive adaptations are introduced.

## 7 Conclusion

Here, we have argued that an evolutionary perspective suggests that the elevated rates of major depressive disorders and anxiety disorders in neurodivergent populations born out in current epidemiological research are best understood as consequences of evolutionary mismatch, rather than as further evidence of inherently “broken brains.” Neurodivergent cognitive traits, plausibly shaped by evolutionary trade‐offs and maintained across generations through mechanisms such as social niche specialisation and balancing selection, interact with modern environmental contexts to overactivate evolved defensive emotional systems. Recognising neurodivergence as part of a natural cognitive diversity, as emphasized by the neurodiversity movement, offers a humane but scientifically grounded perspective in which neurodivergent minds can begin to be appreciated for their strengths, rather than just denigrated for their weaknesses, and elevated risk of conditions like major depressive disorders and anxiety disorders are seen as preventable, rather than predetermined.

Future work could extend this evolutionary framework to other forms of psychopathology that occur at increased rates in neurodivergence populations, including substance use disorders (Lee et al. 2011) and personality disorder diagnoses (Rinaldi et al. 2021). For example, addictive behaviors may plausibly be understood as attempts at self‐regulation or self‐medication in the context of chronically activated affective systems and insufficiently supportive environments, highlighting the need for more context‐sensitive and preventive therapeutic approaches.

## Funding

The authors have nothing to report.

## Conflicts of Interest

The authors declare no conflicts of interest.

## Data Availability

Data sharing not applicable to this article as no datasets were generated or analysed during the current study.
